# A New Tumor Suppressor That Regulates Tissue Architecture

**DOI:** 10.1371/journal.pmed.1000073

**Published:** 2009-05-05

**Authors:** Senthil K. Muthuswamy

**Affiliations:** 1 Ontario Cancer Institute, University of Toronto, Toronto, Ontario, Canada; 2 Cold Spring Harbor Laboratory, Cold Spring Harbor, New York, United States of America

## Abstract

Senthil Muthuswamy discusses a study that characterizes a new tumor suppressor gene, *ductal epithelium-associated RING Chromosome 1*, that is frequently inactivated in breast cancer.

**Linked Research Article**

This Perspective discusses the following new study published in *PLoS Medicine*:

Lott ST, Chen N, Chandler DS, Yang Q, Wang L, et al. (2009) DEAR1 is a dominant regulator of acinar morphogenesis and an independent predictor of local recurrence-free survival in early-onset breast cancer. PLoS Med 6(5): e1000068. doi:10.1371/journal.pmed.1000068

Ann Killary and colleagues describe a new gene that is genetically altered in breast tumors, and that may provide a new breast cancer prognostic marker.

Pathologists use loss of normal tissue architecture as a key criterion to identify and categorize disease states. Epithelia in all glandular structures in vivo have a characteristic organization where they line a central lumen and are involved in absorptive and/or secretory functions. Under some physiological conditions—such as pregnancy, wound healing, and the periodic tissue remodeling that occurs in tissues such as colon—there is a significant increase in the rate of cell proliferation. Although the proliferation rate in these physiological conditions is higher than that seen under disease states, the overall tissue size and structure are maintained. Such maintenance of size/structure occurs because the increased proliferation is tightly coupled with tissue morphogenesis programs, resulting in remodeling of normal tissue architecture.

Aberrant expression of drivers of proliferation, such as growth factors, can induce untimely proliferation of epithelia that results in hyperplastic overgrowth of ducts and alveoli. Despite the increase in cell number, such overgrowth by itself is usually not a cause for concern. However, when the increase in cell number is coupled with atypical changes in tissue *architecture*—such as changes in the organization of epithelia around a lumen, multilayering of the epithelial lining in glandular structures, and changes in cell size or shape—this is usually a cause for concern. While pathways that regulate cell proliferation have been intensely investigated over the past decades, the pathways that regulate cell architecture and tissue organization are poorly understood.

## DEAR1—A New Member of the TRIM Family of Proteins

In this issue of *PLoS Medicine*, Ann Killary and colleagues describe the discovery and characterization of a new tumor suppressor gene, *ductal epithelium–associated RING Chromosome 1* (*DEAR1*) [1], that maps to Chromosome 1p35.1, a region of the chromosome that is associated with loss of heterozygosity in breast and other epithelial cancers [2]. The authors also demonstrate that *DEAR1* is mutated in 13% of primary human breast cancers. Thus *DEAR1* is a frequently inactivated gene in breast cancer.

DEAR1 is a member of the RING-B-box-Coiled-Coiled (RBCC)/tripartite motif (TRIM) family of proteins. TRIM proteins are expressed in response to interferon signaling (for a review see [3]). They have been implicated in a range of biological processes related to innate immunity. In addition, TRIM family members are known to inhibit HIV virus replication and are associated with genetic disorders such as familial Mediterranean fever (a disease associated with increased inflammation). One family member, PML, is an established tumor suppressor that is associated with development of acute promyelocytic leukemia.

All TRIM proteins have a RING domain (see Glossary) in the N terminus [3]. While the RING domain, observed in E3 ubiquitin ligases such as Cbl, was originally shown to play a role in protein ubiquitination [4], recent evidence shows that the RING domain of PML interacts with a SUMO-conjugating enzyme, UBE2I, suggesting that RING domains may also mediate sumoylation [4]. How the TRIM proteins use these domains and what pathways and biological processes they regulate is not yet well understood.

**Glossary**

**RING domain:** A protein domain that consists of a specialized zinc-finger that binds to two atoms of zinc. The RING domain is observed in proteins that regulate the formation of E3 ubiquitin ligase complex.

**E3 ubiquitin ligase:** An enzyme that covalently attaches a small 7.5-kDa protein, ubiquitin, to specific lysine residues on target proteins.

**SUMO:** Small ubiquitin-related modifier. Belongs to a family of ubiquitin-like proteins. There are four SUMO proteins: SUMO1, 2, 3, and 4.

**UBE2I:** Ubiquitin conjugating enzyme 2I.

**Sumoylation:** A type of post-translational modification of proteins that involves reversible covalent modification of specific lysine residues by SUMO.

**Apoptosis:** A programmed cell death process that is used to remove unwanted cells.

**Cell polarity:** A property of a cell to asymmetrically distribute proteins within its intracellular and/or membrane domains.

**CHD5:** Chromodomain helicase DNA binding protein 5.

Most of the TRIM proteins localize to the cytoplasm or the nucleus [3]. DEAR1 is the first member of the TRIM family that localizes to the cell–cell junction, which suggests that the TRIM proteins are involved in processes that involve cell–cell interactions.

## DEAR1 Is a Predictive Biomarker for Early Onset Breast Cancer

Ann Killary and colleagues show that *DEAR1* is expressed in the ductal and glandular epithelia of many adult tissues, including breast, bladder, kidney, prostate, pancreas, and salivary gland. While normal breast epithelia express high levels of *DEAR1*, the authors found that 70% of ductal carcinoma in situ (DCIS) specimens showed a loss or down-regulation of *DEAR1* expression. Such loss or down-regulation suggests a role for DEAR1 during early stages of breast cancer. Consistent with this possible role, the researchers observed a mutation that changes arginine at position 187 to glutamine (R187Q) in both breast tumor and adjacent normal epithelia. This mutation was never seen in normal individuals or in the single nucleotide polymorphism database, suggesting that mutation of *DEAR1* may be an early event that occurs during the initial stages of transformation of normal epithelia.

Alterations in *DEAR1* also show a strong predictive value for future risk of aggressive disease. The authors show that 56% of DCIS stage I or II breast cancers in premenopausal women aged between 25–49 years show a complete loss of *DEAR1* expression. Loss of *DEAR1* expression correlated strongly with family history of breast cancer and with the development of triple negative breast cancers. Together, these observations identify DEAR1 as an excellent predictive biomarker for early onset breast cancers.

## DEAR1 Is Implicated in Epithelial Biology and Carcinoma

When cultured on a bed of extracellular matrix (such as Matrigel), normal mammary epithelial cells form three-dimensional acini-like structures with a layer of polarized epithelial cells surrounding a central hollow lumen. In Killary and colleagues' study, down-regulation of *DEAR1* in normal mammary epithelial cells resulted in formation of aberrant acinar structures with decreased rates of apoptosis and a loss of normal cell polarity. Down-regulation of *DEAR1* did not have any effect on proliferation of these normal mammary epithelial cells, showing that DEAR1 regulates cell architecture pathways *independent* of any effect on cell proliferation.

Breast cancer–derived cells are known to form irregular multiacinar structures in vivo [5]. In the new study, re-expression of *DEAR1* restored these cells' ability to form normal single acini with central empty lumen and a layer of polarized epithelial cells. Here again, re-expression of *DEAR1* did not have a significant effect on the cell proliferation rates, showing that DEAR1 is a critical regulator of 3-D epithelial morphogenesis. Transformation of 3-D organized structures in breast cancer is thought to occur due to a coordinated loss of control over cell proliferation, cell death, and cell polarity pathways. However, several previous studies have shown that cell proliferation pathways are not always coupled to cell polarity and cell death pathways [6]–[9]. Killary and colleagues' study supports the concept that cell polarity and cell proliferation pathways are uncoupled in mammalian epithelial cells. Further analysis will be required to identify the targets of DEAR1 and the mechanism by which DEAR1 regulates cell architecture.

## DEAR1 and CHD5: Two Pieces in a Puzzle

*DEAR1* maps close to another tumor suppressor, *CHD5*, which was recently mapped to Chromosome 1q by a chromosome engineering approach [10]. With the identification of DEAR1 we are beginning to develop a deeper understanding of the molecular basis for the loss of Chromosome 1q in human cancers. While CHD5 regulates cell proliferation pathways by increasing expression of the cell cycle inhibitor p16/ink4a locus, DEAR1 regulates cell architecture. It is possible that CHD5 and DEAR1 represent the two sides of the transformation process, where loss of CHD5 results in aberrant proliferation while loss of DEAR1 results in loss of tissue architecture, and the combination of the events can drive changes in the epithelial tissues that can progress towards cancerous growth. It would be interesting to determine the effect of combined loss of CHD5 and DEAR1 both for clinical prognosis and during transformation of epithelial cells in culture and animal models.

## Abbreviations

DCIS: ductal carcinoma in situ
TRIM: tripartite motif
